# Supplementary material for: Structural assembly of the megadalton-sized receptor for intestinal vitamin B12 uptake and kidney protein reabsorption
Source: Nat Commun. 2018 Dec 6;9:5204. doi: 10.1038/s41467-018-07468-4 (PMC6283879; doi:10.1038/s41467-018-07468-4)
Supplement: Supplementary file 1 — Supplementary Information [file 41467_2018_7468_MOESM1_ESM.pdf]

C. Larsen *et al.*

Supplementary Table 1

Primer name	Primer sequence
Thx_TEV_fwd	5’-CATCACCACAGCCAGAGCGATAAAATTATTCACCTGACTGACG-3’
Thx_TEV_rev	5’-CCAGAGTTTGGAGACTCCTGCTGCGCCGG-3’
AMN_20-357_fwd	5’-GTCTCCAAACTCTGGGTCCCCAACACGGACTTC-3’
AMN_20-357_rev	5’-CGCCGAGCTCGAATTTCAGGAGCTGCCCCAG-3’
Cubilin_26-135_fwd	5’-GAAGGAGATATACATATGGGAGAACTTGAGCTGCA-3’
Cubilin_26-135_rev	5’-TCCAATTGAGATCTGTCAAACCTTTTTGTCAACAGTCTGC-3’
AMN_1-454_fwd	5’-GAAGAATTCAATGGGCGTCCTGGGCCGG-3’
AMN_1-454_rev	5’-GAGTCTAGATCAGGCCTCGGCCTCGGC-3’
AMN_L59P_fwd	5’-TCAGTCCCGGTGCAAGAAGGTCACGCCGTCTCAGAC-3’
AMN_L59P_rev	5’-TTGCACCGGGACTGACACCATCTTGTCCGCCGGG-3’
AMN_M69K_fwd	5’-TCAGACAAGCTCCTGCCGCTGGATGGGGAACTCG-3’
AMN_M69K_rev	5’-CAGGAGCTTGTCTGAGACGGCGTGACCTTCTTGCAC-3’
AMN_C234F_fwd	5’-GGCCGCTTCCCCCAGGCCGCCTGCCACAGCGCC-3’
AMN_C234F_rev	5’-CTGGGGGAAGCGGCCGCCCAGGGGCTGGAGCAG-3’
AMN_G254E_fwd	5’-CTCTGTGAAGCCGTTGTGTTGCTGACCCACGGCC-3’
AMN_G254E_rev	5’-AACGGCTTCACAGAGGTCACAGCACTGCCCCTGG-3’
SLIM primers:	
AMN N35Q_fwd	5’-CAGAACCGGACCCCGTGC-3’
AMN N35Q_fwd tailed	5’-GTCGCAGCCCAATGGAGCCAGAACCGGACCCCGTGC-3’
AMN N35Q_rev	5’-GTCGAAGTCCGTGTTGGG-3’
AMN N35Q_rev tailed	5’-GCTCCATTGGGCTGCGACGTCGAAGTCCGTGTTGGG-3’
AMN S37A_fwd	5’-CCGTGCGCCGGCGGCGCC-3’
AMN S37A_fwd tailed	5’-AACTGGGCACAGAACCGGACCCCGTGCGCCGGCGGCGCC-3’
AMN S37A_rev	5’-GGCTGCGACGTCGAAGTC-3’
AMN S37A_rev tailed	5’-GGTCCGGTTCTGTGCCCAGTTGGCTGCGACGTCGAAGTC-3’
AMN T41I_fwd	5’-GGCGGCGCCGTTGAGTTC-3’
AMN T41I_fwd tailed	5’-AACCGGATCCCGTGCGCCGGCGGCGCCGTTGAGTTC-3’
AMN T41I_rev	5’-CTGGCTCCAGTTGGCTGC-3’
AMN T41I_rev tailed	5’-GGCGCACGGGATCCGGTTCTGGCTCCAGTTGGCTGC-3’
AMN_T41I_fwd	5’-AACCGGATCCCGTGCGCCGGCGGCGCCGTTGAG-3’
AMN_T41I_rev	5’-GCACGGGATCCGGTTCTGGCTCCAGTTGGCTGCG-3’
AMN_T41I_fwd	5’-AACCGGATCCCGTGCGCCGGCGGCGCCGTTGAG-3’
AMN_T41I_rev	5’-GCACGGGATCCGGTTCTGGCTCCAGTTGGCTGCG-3’

Supplementary Table 2

**Missense mutations of AMN causing Imerslund-Gräsbeck syndrome**		
**Substitution**	**Position**	**Reference**
Thr → Ile	41	Tanner, S. M. *et al*. Nat. Genet. 33, 426–429 (2003)[1].
Leu → Pro	59	Tanner, S. M. *et al*. Orphanet J Rare Dis 7, 56 (2012)[2].
Met → Lys	69	Montgomery, E. *et al*. BMC Med. Genet. 16, 35 (2015)[3].
Cys → Phe	234	Luder, A. S. *et al.* J. Inherit. Metab. Dis. 31 Suppl 3, 493–496 (2008)[4].
Gly → Glu	254	Tanner, S. M. *et al*. Orphanet J Rare Dis 7, 56 (2012)[2].

Supplementary Figure 1

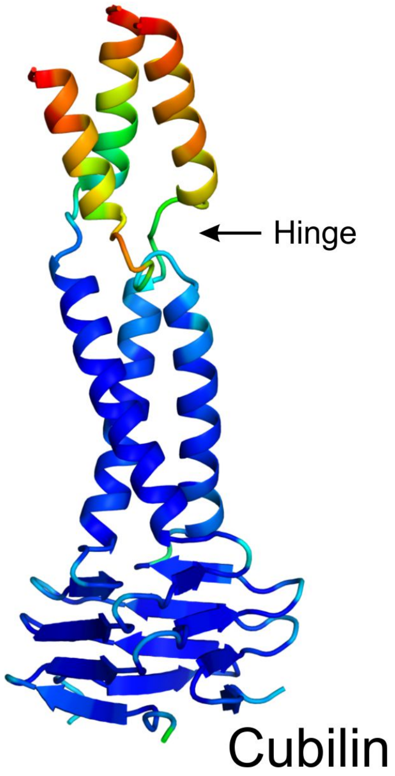

Supplementary Figure 1: B-factors indicate that the cubilin hinge-region introduces flexibility to the cubam receptor. Cartoon representation of cubilin (residue 36-135) coloured according to B-factors (blue – low, red – high). A dramatic increase in B-factors in observed in the region following the hinge suggests that the hinge introduces flexibility to the cubam receptors.

Supplementary Figure 2

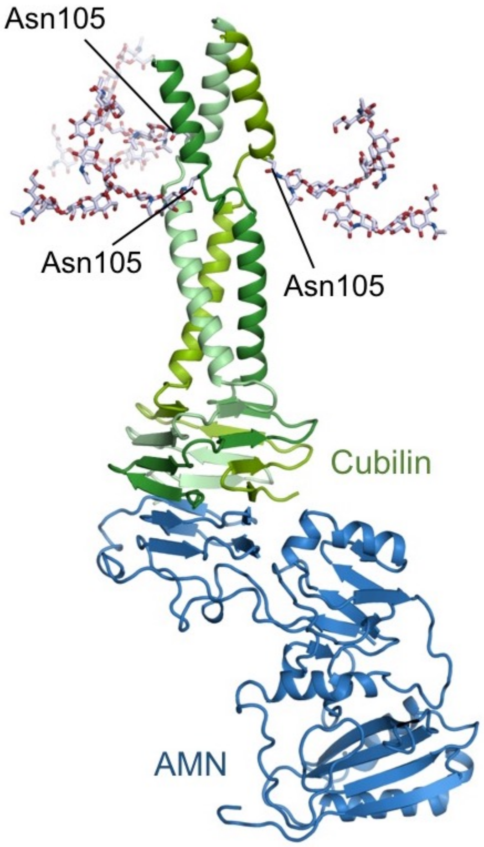

Supplementary Figure 2: N-linked glycosylation of cubilin. The N-terminal region of cubilin contains a sequence motif for N-linked glycosylation of Asn105. Mature N-linked glycosylations can be modelled on cubilin Asn105 residues with only minor rearrangements of the Asn105 sides chains.

Supplementary Figure 3

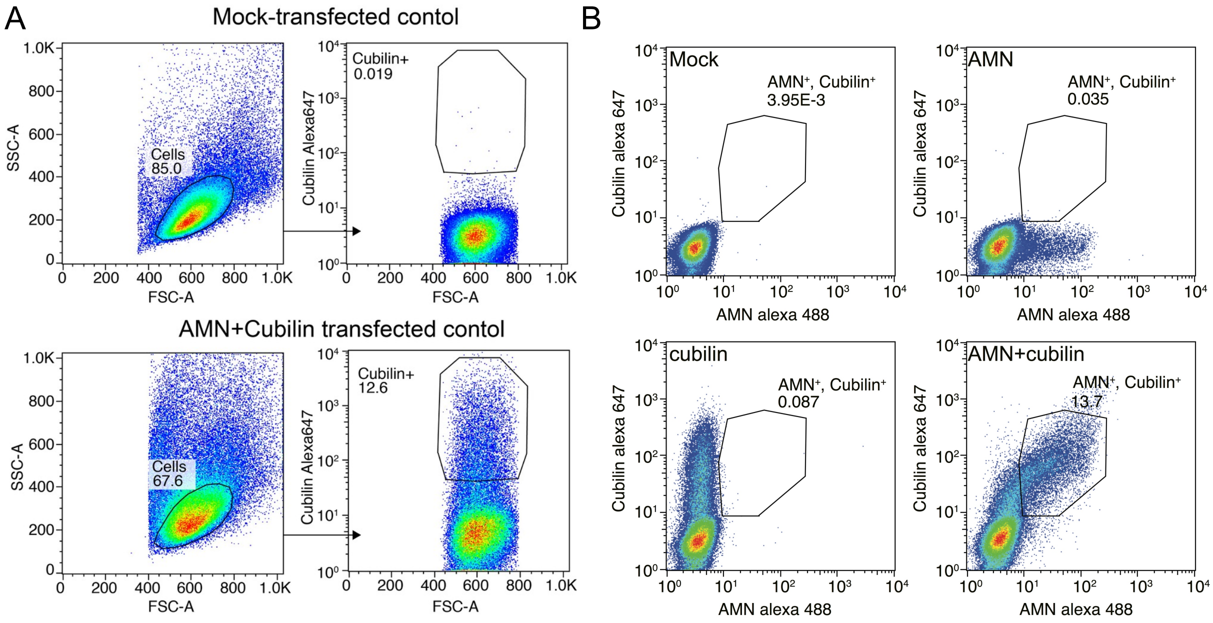

Supplementary figure 3: **Surface expression of cubilin in transiently transfected CHO K1 cells. A.** Gating strategy of Cubilin+ CHO K1 cells in transiently transfected cells co-transfected with AMN and cubilin cDNA. **B.** Representative intra-cellular flow cytometric analysis of transient transfection efficiency of AMN and cubilin co-transfection. Cells were gated in FSC-A and SSC-A and replotted as AMN vs cubilin.

Supplementary Figure 4

A

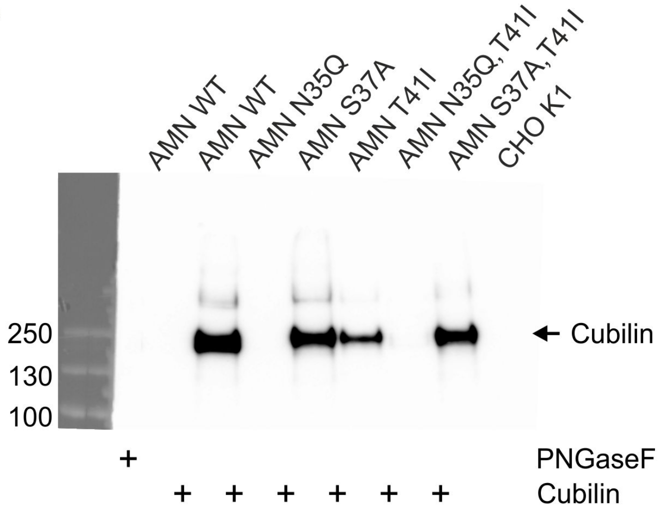

B

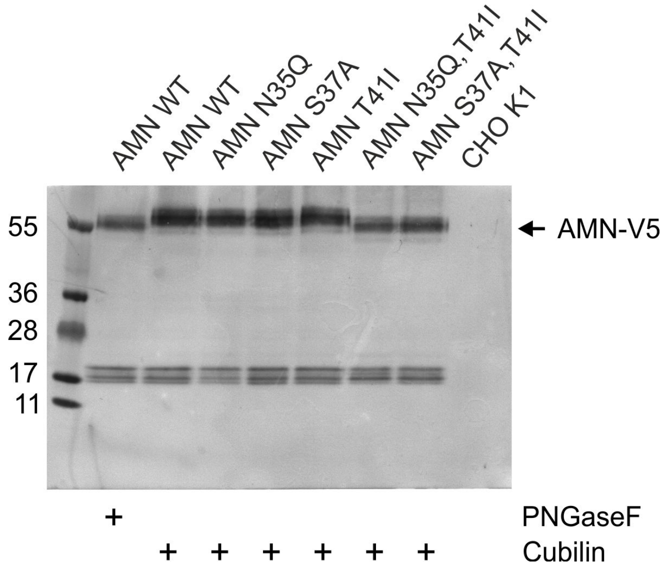

Supplementary figure 4: Immunoprecipitation of cubilin with wild type or mutant forms of AMN-V5. A. Uncropped western blot from Figure 5C (top). The blot was visualized using rabbit polyclonal anti-rat cubilin antibody followed by Horse-radish peroxidase conjugated goat polyclonal anti-rabbit IgG. **B.** Uncropped western blot from Figure 5C (bottom). The blot was visualized using mouse monoclonal anti-V5 alkaline phosphatase (AP) conjugated antibody.

Supplementary Figure 5

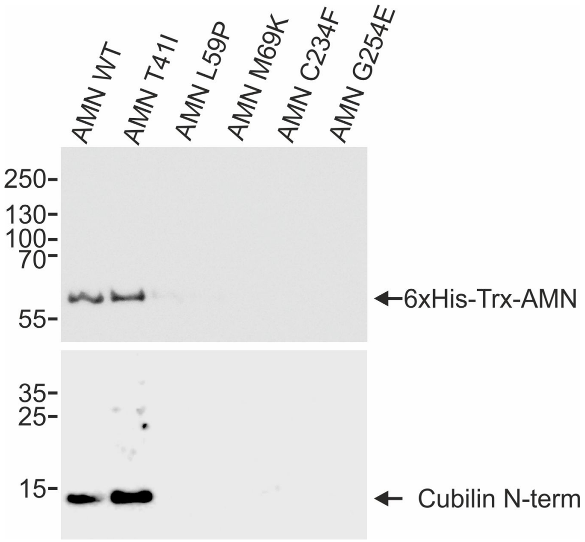

Supplementary figure 5: Western blot of eluted fractions from Ni-affinity chromatography of AMN(20-357) and cubilin(26-135) expressed in *E. coli*. The western blots were performed using a mouse monoclonal anti-AMN (AMN 17-44-3) antibody (upper blot) and a mouse monoclonal anti-cubilin (cubilin 17-44-5) antibody (lower blot) using a horse-radish peroxidase-conjugated goat polyclonal anti-mouse IgG as secondary antibody.

Supplementary references

1. Tanner, S. M. *et al.* Amnionless, essential for mouse gastrulation, is mutated in recessive hereditary megaloblastic anemia. *Nat. Genet.* **33,** 426–429 (2003).
2. Tanner, S. M., Sturm, A. C., Baack, E. C., Liyanarachchi, S. & la Chapelle, de, A. Inherited cobalamin malabsorption. Mutations in three genes reveal functional and ethnic patterns. *Orphanet J Rare Dis* **7,** 56 (2012).
3. Montgomery, E. *et al.* Novel compound heterozygous mutations in AMN cause Imerslund-Gräsbeck syndrome in two half-sisters: a case report. *BMC Med. Genet.* **16,** 35 (2015).
4. Luder, A. S., Tanner, S. M., la Chapelle, de, A. & Walter, J. H. Amnionless (AMN) mutations in Imerslund-Gräsbeck syndrome may be associated with disturbed vitamin B12 transport into the CNS. *J. Inherit. Metab. Dis.* **31 Suppl 3,** 493–496 (2008).
