# Supplementary material for: Structural assembly of the megadalton-sized receptor for intestinal vitamin B12 uptake and kidney protein reabsorption
Source: Nat Commun. 2018 Dec 6;9:5204. doi: 10.1038/s41467-018-07468-4 (PMC6283879; doi:10.1038/s41467-018-07468-4)
Supplement: Supplementary file 3 — Reporting Summary [file 41467_2018_7468_MOESM3_ESM.pdf]

nature research

Corresponding author(s): *Double-blind peer review submissions: write DBPR and your manuscript number here instead of author names.*

Reporting Summary

Nature Research wishes to improve the reproducibility of the work that we publish. This form provides structure for consistency and transparency in reporting. For further information on Nature Research policies, see Authors & Referees and the Editorial Policy Checklist.

Statistical parameters

When statistical analyses are reported, confirm that the following items are present in the relevant location (e.g. figure legend, table legend, main text, or Methods section).

n/a	Confirmed	
☒	☐	The exact sample size (*n*) for each experimental group/condition, given as a discrete number and unit of measurement
☐	☒	An indication of whether measurements were taken from distinct samples or whether the same sample was measured repeatedly
☒	☐	The statistical test(s) used AND whether they are one- or two-sided<br>*Only common tests should be described solely by name; describe more complex techniques in the Methods section.*
☒	☐	A description of all covariates tested
☒	☐	A description of any assumptions or corrections, such as tests of normality and adjustment for multiple comparisons
☒	☐	A full description of the statistics including central tendency (e.g. means) or other basic estimates (e.g. regression coefficient) AND variation (e.g. standard deviation) or associated estimates of uncertainty (e.g. confidence intervals)
☒	☐	For null hypothesis testing, the test statistic (e.g. *F*, *t*, *r*) with confidence intervals, effect sizes, degrees of freedom and *P* value noted<br>*Give P values as exact values whenever suitable.*
☒	☐	For Bayesian analysis, information on the choice of priors and Markov chain Monte Carlo settings
☒	☐	For hierarchical and complex designs, identification of the appropriate level for tests and full reporting of outcomes
☒	☐	Estimates of effect sizes (e.g. Cohen's *d*, Pearson's *r*), indicating how they were calculated
☐	☒	Clearly defined error bars<br>*State explicitly what error bars represent (e.g. SD, SE, CI)*

Our web collection on statistics for biologists may be useful.

Software and code

Policy information about availability of computer code

Data collection	mxCuBE v2<br>Appion 3.2<br>Leginon 3.2<br>BD Cell Quest<br>LAS-3000 lite
Data analysis	XDS version Jan 26, 2018<br>PHENIX dev-2614<br>COOT 0.8.9<br>Relion 2.0<br>FlowJo software for Mac ver. 10.4

For manuscripts utilizing custom algorithms or software that are central to the research but not yet described in published literature, software must be made available to editors/reviewers upon request. We strongly encourage code deposition in a community repository (e.g. GitHub). See the Nature Research guidelines for submitting code & software for further information.

Data

Policy information about availability of data

All manuscripts must include a data availability statement. This statement should provide the following information, where applicable:

- Accession codes, unique identifiers, or web links for publicly available datasets
- A list of figures that have associated raw data
- A description of any restrictions on data availability

Atomic structure factors and coordinates have been deposited at the Protein Data Bank under accession number 6GJE.
Figure 5: Flow cytometry data are available upon request.

Field-specific reporting

Please select the best fit for your research. If you are not sure, read the appropriate sections before making your selection.

☒ Life sciences ☐ Behavioural & social sciences ☐ Ecological, evolutionary & environmental sciences

For a reference copy of the document with all sections, see nature.com/authors/policies/ReportingSummary-flat.pdf

Life sciences study design

All studies must disclose on these points even when the disclosure is negative.

Sample size	Not applicable. Manuscript only contains in vitro data on recombinant protein and recombinant cell lines
Data exclusions	Not applicable
Replication	In vitro data on transient transfected cells was performed in triplicates with individual transfections. Experiments was repeated twice
Randomization	Not applicable
Blinding	Not applicable

Reporting for specific materials, systems and methods

Materials & experimental systems

n/a	Involved in the study
☒	☐ Unique biological materials
☐	☒ Antibodies
☐	☒ Eukaryotic cell lines
☒	☐ Palaeontology
☒	☐ Animals and other organisms
☒	☐ Human research participants

Methods

n/a	Involved in the study
☒	☐ ChIP-seq
☐	☒ Flow cytometry
☒	☐ MRI-based neuroimaging

Antibodies

Antibodies used	anti V5 agarose beads (Sigma, clone V5-10)<br>mouse monoclonal anti V5 alkaline phosphatase (AP) conjugated antibody (Thermo Fischer, cat no. R96225)<br>rabbit polyclonal anti rat cubilin antibody (Moestup et. al. JBC, 273, 5235-5242. (1998))<br>mouse anti-AMN antibody (AMN 17-44-3, own)<br>mouse anti-cubilin antibody (cubilin 17-44-5, own)
Validation	AMN 17-44-3 and cubilin 17-44-5 were validated in western blots against purified porcine CUBAM.

Eukaryotic cell lines

Policy information about cell lines

Cell line source(s)	CHO K1 cells were obtained from Invitrogen (Thermo Scientific)
Authentication	CHO K1 cells were not authenticated
Mycoplasma contamination	CHO K1 cell lines were not tested for mycoplasma contamination in relation to this study
Commonly misidentified lines (See ICLAC register)	None

Flow Cytometry

Plots

Confirm that:

☒ The axis labels state the marker and fluorochrome used (e.g. CD4-FITC).

☒ The axis scales are clearly visible. Include numbers along axes only for bottom left plot of group (a 'group' is an analysis of identical markers).

☒ All plots are contour plots with outliers or pseudocolor plots.

☒ A numerical value for number of cells or percentage (with statistics) is provided.

Methodology

Sample preparation	adherent cells were harvested from culture dishes using accutase (Sigma)
Instrument	BD FACSCalibur™ flow cytometer (BD Bioscience)
Software	Flow cytometric data was collected using BD Cell Quest and analyzed using FlowJo software for Mac ver. 10.4
Cell population abundance	not applicable
Gating strategy	Live cells were gated in SSC-A and FSC-A and replotted in a contour plot showing Cubilin expression vs FSC-A. Correct gate for Cubilin expressing cells (Cubilin+) was set based on non-cubilin expressing control cells.

☒ Tick this box to confirm that a figure exemplifying the gating strategy is provided in the Supplementary Information.
